# Efficient Microwave-Assisted Synthesis of Ionic Esterified Amino Acids

**DOI:** 10.3390/molecules16108733

**Published:** 2011-10-19

**Authors:** Ricardo Cerón-Camacho, Jorge Aburto, Luisa E. Montiel, Eugenio A. Flores, Frisia Cuellar, Rafael Martínez-Palou

**Affiliations:** Dirección de Investigación y Posgrado, Instituto Mexicano del Petróleo, Eje Central Lázaro Cárdenas 152, 07730 Mexico City, Mexico

**Keywords:** amino acids, esterification, microwave, parallel synthesis

## Abstract

In this work, an efficient microwave-assisted methodology for the esterification of unprotected α-amino acids is described. Ionic esterified amino acids were synthesized in satisfactory yields in a facile one-pot solventless protocol from unprotected amino acids and alcohols under acid catalysis (MsOH or *p*-TsOH) to afford the pure products after a simple work-up procedure. This procedure can also be extended to the preparation of long and short chain alkyl and benzyl esters.

## 1. Introduction

Microwave technology has become a powerful tool in organic synthesis which is able to grant access to a wide range of organic compounds in a very simple, swift and efficient way The use of microwave dielectric heating have been shown to dramatically reduce processing time and often leading to high purities and better yields to products as compared to conventional methods [1,2,3,4].

Parallel synthesis of combinatorial libraries can be described as synthetic sequences using an ordered array of spatially separated reaction vessels under the same reaction conditions which generally yield a more or less extensive library of compounds [5]. Parallel synthesis has been reported under both conventional heating conditions and, more recently, under microwave irradiation for combinatorial chemistry [6]. In this regard, microwaves can allow a quick and simple optimization of reaction conditions including time, suppression of byproducts, improved yields which can significantly simplify the synthesis and/or development of novel organic compounds in an efficient fashion.

The synthesis of carboxylic esters is one of the most fundamental protocols for producing natural and synthetically useful chemicals in peptide chemistry [7]. Particularly, long alkyloyl amino acids are important compounds due to their nutritional [8] and surfactant properties [9]. Some of them also show antisickling activity [10], protective properties against microorganism growth, as well as in the preservation of perishable food products [11].

Synthetic routes to prepare such compounds are rather limited to date, in spite of the wide applications of amino acid esters. These methods often rely on the use of the alcohol components in the liquid phase (as both solvent and reagent) so they are therefore not readily applicable to long chain solid alcohols [12]. Furthermore, most reported protocols to date lack green credentials and either require the presence of strong acids such as HCl or H_2_SO_4_ [13,14], hazardous reagents including *p*-toluenesulfonyl chloride [15], diazomethane [16] or orthoesters [17]. In some other cases, coupling agents (e.g., carbodiimide) [18] need to be employed or poorly atom efficient protocols with several reaction steps (protection, esterification and deprotection) as well as the use of expensive *N*-protected amino acids starting materials [19,20,21]. Many of such protocols also have a limited scope [22]. Comparatively, few heterogeneous catalysts including ionic liquids have been described as efficient systems to prepare short alkyl amino acid esters via esterification of amino acids [23,24] but these approaches have a limited applicability (e.g., nitrogen atmosphere) and/or suffer from many of the aforementioned drawbacks.

In this work, we report a one-pot, solventless and highly versatile microwave-assisted methodology for the esterification and simultaneous salt formation of unprotected α-amino acids using long chain alcohols and simple organic acid catalysts [methanesulfonic acid (MsOH) and *p*-toluenesulfonic acid (*p*-TsOH)] to obtain ionic amino acids ester with organic anions. The proposed approach could be efficiently extended to the synthesis of long and short chain alkyl and aryl esters. To the best of our knowledge, this is the first report of a general procedure for the esterification of unprotected amino acids under microwave irradiation, despite other investigations related to the esterification of amino acids under microwave irradiation [19,20,21,22].

## 2. Results and Discussion

### 2.1. Microwave-Assisted Parallel Synthesis of Ionic-Esterified Amino Acids

The esterification reaction of carboxylic acids under microwave irradiation has been widely study [25,26,27], however the esterification of amino acids is comparatively more difficult to that of ordinary carboxylic acids as a consequence of their zwitterionic structure.

After several experiments to establish the optimum conditions (data not shown), a versatile procedure was obtained for the parallel synthesis of ionic-esterified α-amino acids from unprotected amino acids, alcohols and acid catalysts (MsOH or *p*-TsOH) under microwave irradiation. Reactions were carried out employing glycine (entries 1–8), D-alanine (entries 9–14) and DL-tryptophan (entries 15–21, Table 1).

**Table 1:** Parallel synthesis of ionic-esterified amino acids under microwave irradiation. 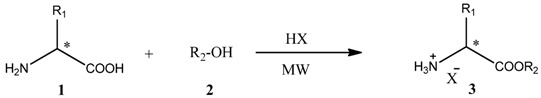

Entry	R_1_	R_2_	X^−^	Product	Yield (%) ^a^
1	H	*i*-C_3_H_7_	MsO	**3a**	75
2	H	C_4_H_9_	MsO	**3b**	78
3	H	C_8_H_17_	*p*-TsO	**3c**	63
4	H	C_10_H_21_	MsO	**3d**	78
5	H	C_12_H_25_	*p*-TsO	**3e**	62
6	H	C_14_H_29_	MsO	**3f**	79
7	H	C_16_H_33_	MsO	**3g**	78
8	H	C_18_H_37_	MsO	**3h**	84
9	Me	Bz	MsO	**3i**	76
10	Me	C_2_H_5_	*p*-TsO	**3j**	73
11	Me	C_8_H_17_	*p*-TsO	**3k**	21
12	Me	C_10_H_21_	MsO	**3l**	74
13	Me	C_12_H_25_	*p*-TsO	**3m**	24
14	Me	C_14_H_29_	MsO	**3n**	76
15	3-methylene-1*H*-indole (3MIn)	C_8_H_17_	MsO	**3o**	77
16	3MIn	C_10_H_21_	MsO	**3p**	74
17	3MIn	C_12_H_25_	MsO	**3q**	75
18	3MIn	C_14_H_29_	MsO	**3r**	75
19	3MIn	C_16_H_33_	MsO	**3s**	78
20	3MIn	C_18_H_37_	MsO	**3t**	74
21	3MIn	C_18_H_37_	*p*-TsO	**3u**	62

^a^ Isolated yields.

Table 1 proves ionic-esterified amino acids (ionic liquids analogues) could be obtained in one-step by simultaneous esterification of the carboxylic group and protonation of the amine group (with subsequent formation of the organic salt end). The ionic product is more stable than the neutral and consequently inter- and intramolecular reactions could be avoided in our proposed approach. Due to their ionic character, amino acids interact very efficiently with microwave irradiation providing a fast and homogeneous increase in temperature.

Simultaneous cooling with compressed air (5 bar) was applied in order to prevent the strong exotherm in the synthesis, avoiding overheating and abrupt temperature overshoot (presented when reaction was carried out without air cooling) and allows a higher level of irradiation power at the established temperature [28,29].

**Figure 1:**
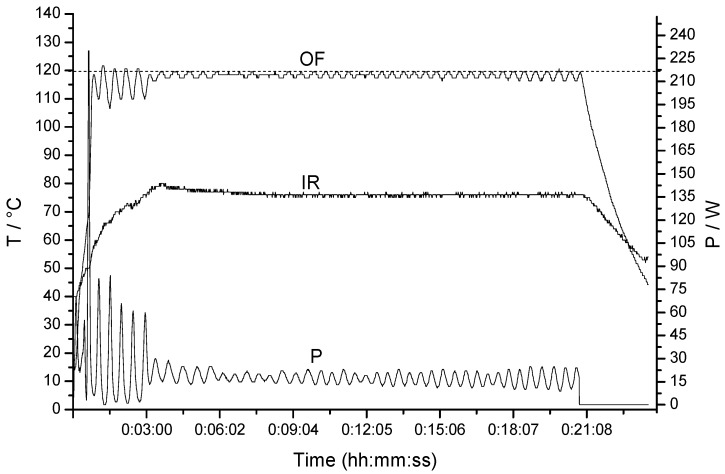
FO/IR temperature (T) and power (P) profiles for the reaction to obtain compound **3b**.

In these reactions the temperature profiles were obtained simultaneously by both external IR and external FO sensor [30]. Due to the strong microwave absortivity of our ionic products and the delay experience in monitoring temperature on the outer surface of a heavy-walled vessel and especially in our experiments with simultaneous cooling, the magnetron output power was controlled by the most precise internal FO sensor (IR as slave). In all experiments significant differences between FO and IR measurement (~40–50 °C) were observed, as can be seen as example in FO/IR temperature (T) and power (P) profiles in the synthesis of the compound **3b** (Figure 1).

Particularly related to this work, our efforts have been especially focused in the synthesis of ionic amphiphilic amino acids with long alkyl chains (C_8_–C_18_, Table 1). However, it is worth pointing out that the proposed versatile approach can also be extended to the preparation of short chain (entries 1, 2 and 10, Table 1) and benzyl (entry 9, Table 1) esterified ionic amino acids.

Good yields to products could be obtained for all investigated amino acids and alcohols, regardless of their structure and composition, except for products **3k** and **3m** where ionic product from acid-base reaction without esterification, was curiously the main product. Products were obtained in acceptable purities (>95% as seen by NMR) after a simple workup comprising of crystallization via EtOH addition and subsequent washing with ethyl ether.

MsOH and *p*-TsOH were found to be suitable catalysts for the reactions under the investigated reaction conditions but yields were higher for the case of MsOH as compared to those obtained with *p*-TsOH. An additional advantage of these reactions is that a high alcohol excess was not required to obtained high yields to products as compared to most literature reported protocols [19,20,21,23]. The data collected shows that no significant racemization (ratio ~ D:L, 5:1) take place during the synthesis for products obtained from D-alanine.

### 2.2. Amphiphilic Properties of Ionic Amino Acids with Long Alkyl Chains

The critical miscellar concetration (CMC) is an important characteristic of a surfactant. Besides the essential contribution of the hydrophobic interactions, the micellization of ionic surfactants in aqueous solutions is influenced considerably by the electrostatic interactions between the ionized head-groups and their interactions with the surrounding counterions and water molecules.

The surfactant CMC determination is indeed done in an aqueous system where the surfactant forms micelles with polar heads oriented toward the aqueous medium. The amphiphilic properties of water soluble long alkyl chain (C8–C14) synthesized compounds were determined for measuring the CMCs for compounds **3c–3f** obtained from glycine. CMCs were determined by two methods, using the classical method by interfacial tensiometry and by Steady-state fluorescence measurements [31]. The results are showed in Table 2.

**Table 2:** CMCs values of **3c–3f** determined by interfacial tensiometry (IT) and by Steady-state fluorescence (F) measurements.

Compound	CMC (ppm) by IT	CMC (ppm) by F
**3c**	151.2	151.4
**3d**	75.7	60.0
**3e**	9.8	12.1
**3f**	2.9	4.0

As can be observed in Table 2, good agreement in the values was obtained by both methods. The carbon chain length of the hydrophobe was found to be a determining factor in the value of CMC, for the prepared series of amino acid esters surfactants, we found that was a marked decline in CMC values with increase the number of carbon atoms in chain length. CMC for C_8_ is higher than C_10_, whereas C_10_ is higher than C_12_. The CMC values decreases as hydrophobic group size increases. Similar behavior is typically for other surfactants series, such as alkylpolyglucosydes [32,33] and *N*-acetylated cationic surfactants [34]. These results point out that these compounds could be employed as emulsifiers for oilfield applications.

## 3. Experimental

### 3.1. General

All Aldrich reagents were used without previous purification. *p*-TsOH was dehydrated by heating (100 °C) y vacuum during 4 h. Melting points were measured in a Fisher Scientific apparatus with a 300 °C thermometer. ^1^H-NMR (300 MHz) and ^13^C-NMR (75.4 MHz) spectra were obtained with a Varian-Gemini-300 equipment using TMS as internal standard and the solvent specified in each case at room temperature, as shown below. IR spectra were recorded on a Nicolet spectrometer Nexus 470 FT-IR (KBr powder, diffuse reflectance mode). Microwave reaction were performed using a commercially available mono-mode microwave, Monowave 300 manufactured by Anton Paar [35], employing a 10 mL Pyrex vial in a closed vessel mode. Reactions were carried out with simultaneous cooling with compressed air (5 bar) and stirring at a fixed rate of 800 rpm. The reaction temperatures were monitored by both, an external infrared sensor (IR) and by an internal fiber-optic (FO) temperature probe (ruby thermometer) protected by a borosilicate immersion well inserted directly in the reaction mixtures. The magnetron output power was controlled by the FO probe (IR as slave) and heating rates was controlled by selecting the “as-fast-as-possible” mode. Pressure sensing is achieved by a hydraulic sensor integrated in the swiveling cover of the instrument. Critical Micellar Concentrations (CMC) were determined by tensiometry and by fluorometry methods. Different concentrations of each sample were prepared and the surface tension at 25 °C was measured using a platinum ring on a tensiometer DuNoüy. Several concentrations were prepared by diluting the stock solution with distilled water (interfacial tension = 72 dyne/cm) to the appropriate concentration to be used in the determination of the critical micelle concentration (CMC). The surfactants CMC determinations by fluorometric spectroscopy were performed on a RF-5301PC Shimadzu spectrofluorometer equipped with a 150 W Xe lamp and a cell temperature controller using pyrene as a fluorophore probe. The emission spectra of pyrene and assays with surfactants were recorded between 350 and 600 nm using a λ_exc_ of 334 nm at 25 °C. The calculations for CMC were determined from the emission spectra of each surfactant as described before [31].

### 3.2. General Procedure for Microwave-Assisted Synthesis of Ionic-Esterified Amino Acids Salts

In a reaction vessel provided with reflux condenser, alcohols (5 mmol) were heated for 30 s at 70 °C with magnetic stirring under microwave irradiation at 70 W, to better homogenize compounds at the bottom of the microwave tube in the case of solid alcohols. The corresponding amino acids (2.5 mmol) and the catalyst (MsOH or *p-*TsOH, 6 mmol) were then added and the mixture was irradiated for 20 min under simultaneous cooling with air (20 psi). Upon reaction completion, the final obtained product was diluted with hot ethanol (10 mL) and ether (20 mL). The mixture was allowed to stand overnight at 5 °C. A mixture of the corresponding amino acid (2.5 mmol), alcohol (5.0 mmol) and the catalyst (MsOH or *p-*TsOH, 6 mmol) were placed in a 10 mL Pyrex reaction vessel equipped with a magnetical stir bar and fitted with an immersion well for the ruby thermometer. The reaction mixture was first irradiated 30 s at 70 °C and then for 20 min under simultaneous cooling with compressed air (5 bar). Upon reaction completion, the final obtained product was diluted with hot ethanol (10 mL) and ether (20 mL). The mixture was allowed to stand 1 h at 5 °C. The precipitate product was vacuum-filtered, washed with ethyl ether and dried under vacuum. Products were characterized by IR, ^1^H and ^13^C-NMR.

*2-Oxo-2-(propan-2-yloxy)ethanaminium methanesulfonate *(**3a**). White solid; Mp. 99–104 °C; ^1^H-NMR (δ, DMSO-*d*_6_): 8.36 (ws), 7.46 (d, *J* = 7.8 Hz, 1H), 7.36 (d, *J* = 7.9 Hz, 1H), 7.21 (dd, *J* = 6.8, 2.1 Hz, 1H), 7.05 (td, *J* = 6.8, 2.1 Hz, 1H), 6.97 (td, *J* = 6.8, 1.0 Hz, 1H), 4.17 (s, 1H), 4.01 (m, 2H), 3.27 (m, 2H), 2.46 (s, 3H), 1.45 (qi, *J* = 6.7 Hz, 2H), 1.20 (m, 10H), 0.85 (t, *J* = 7.1 Hz, 3H); ^13^C-NMR: 167.5, 135.4, 134.4 124.9, 122.9, 119.2, 116.7, 115.9, 109.7, 104.4, 77.4, 76.9, 76.5, 63.8, 50.9, 29.42, 26.8, 26.7 25.9, 24.4, 23.3, 20.3, 12.0; IR, RD-KBr (cm^−1^): 3315, 3055, 2983, 2935, 1740, 1597, 1516, 1493, 1439, 1464, 1383, 1356, 1338, 1290, 1205, 1176, 1119, 1072, 1045, 957, 899, 779, 742, 557.

*2-(Butyloxy)-2-oxoethanaminium methanesulfonate *(**3b**). White solid; Mp. 117–119 °C; ^1^H-NMR (δ, DMSO-*d*_6_): 8.36 (ws), 7.46 (d, *J* = 7.8 Hz, 1H), 7.36 (d, *J* = 7.9 Hz, 1H), 7.21 (dd, *J* = 6.8, 2.1 Hz, 1H), 7.05 (td, *J* = 6.8, 2.1 Hz, 1H), 6.97 (td, *J* = 6.8, 1.0 Hz, 1H), 4.17 (s, 1H), 4.01 (m, 2H), 3.27 (m, 2H), 2.46 (s, 3H), 1.45 (qi, *J* = 6.7 Hz, 2H), 1.20 (m, 10H), 0.85 (t, *J* = 7.1 Hz, 3H); ^13^C-NMR: 167.5, 1354.5, 134.4 124.9, 122.9, 119.2, 116.7, 115.9, 109.7, 104.4, 77.4, 76.9, 76.5, 63.8, 50.9, 29.42, 26.8, 26.7 25.9, 24.4, 23.3, 20.3, 12.0; IR, RD-KBr (cm^−1^): 3421, 3024, 2966, 2881, 1747, 1610, 1506, 1435, 1319, 1207, 1169, 1045, 906, 864, 781, 557.

*2-(Octyloxy)-2-oxoethanaminium p-toluenesulfonate* (**3c**). White solid; Mp. 200–201 °C; ^1^H-NMR (δ, CD_3_OD): 8.24 (ws), 7.73 (d, *J* = 7.7 Hz, 2H), 7.22 (d, *J* = 7.9 Hz, 2H), 3.79 (s, 2H), 3.56 (t, *J* = 7.0 Hz, 2H), 2.37 (s, 3H), 1.54 (m, 2H), 1.29 (m, 10H), 0.89 (t, *J* = 7.0 Hz, 3H); ^13^C-NMR: 167.7, 140.8, 140.0, 128.2, 125.0, 77.4, 76.9, 76.5, 39.2, 20.2; IR, RD-KBr (cm^−1^): 3460, 3061, 2954, 2848, 1755, 1595, 1503, 1443, 1242, 1161, 1038, 1012, 928, 860.

*2-(Decyloxy)-2-oxoethanaminium methanesulfonate* (**3d**). White solid; Mp. 67–68 °C; ^1^H-NMR (δ, CD_3_OD): 8.34 (ws), 4.24 (t, *J* = 6.7 Hz, 2H), 3.83 (s, 2H), 2.72 (s, 3H), 1.69 (qi, *J* = 6.7 Hz, 2H), 1.29 (m, 14H), 0.89 (t, *J* = 6.9 Hz, 3H); ^13^C-NMR: 168.3, 67.2, 40.9, 39.4, 32.8, 30.5, 30.4, 30.2, 30.1, 26.7, 23.6, 14.5; IR, RD-KBr (cm^−1^): 3454, 3016, 2956, 2924, 1749, 1593, 1500, 1468, 1421, 1379, 1207, 1192, 1051, 904, 785.

*2-(Dodecyloxy)-2-oxoethanaminium p-toluenesulfonate* (**3e**). White solid; Mp. 195–196 °C; ^1^H-NMR (δ, CD_3_OD): 8.27 (ws), 7.73 (d, *J* = 7.7 Hz, 2H), 7.22 (d, *J* = 7.2 Hz, 2H), 4.19 (t, *J* = 6.8 Hz, 2H), 3.78 (s, 2H), 2.37 (s, 3H), 1.66 (qi, *J* = 6.7 Hz, 2H), 1.28 (m, 18H), 0.89 (t, *J* = 6.9 Hz, 3H); ^13^C-NMR: 167.6, 140.9, 139.8, 128.0, 124.9, 77.4, 76.9, 76.6, 39.0, 28.8, 28.7, 28.5, 19.9; IR, RD-KBr (cm^−1^): 3467, 3223, 3059, 2954, 2920, 1751, 1595, 1506, 1443, 1242, 1161, 1124, 1038, 1012, 926, 860.

*2-(Tetradecyloxy)-2-oxoethanaminium methanesulfonate* (**3f**). White solid; Mp. 78–79 °C. ^1^H-NMR (δ, CD_3_OD): 8.32 (ws), 4.23 (t, *J* = 6.7 Hz, 2H), 3.83 (s, 2H), 2.71 (s, 3H), 1.69 (qi, *J* = 6.7 Hz, 2H), 1.28 (m, 22H), 0.89 (t, *J* = 7.0 Hz, 3H); ^13^C-NMR: 168.5, 67.3, 40.9, 40.7, 39.46, 32.9, 30.7, 30.68, 30.63, 30.5, 30.4, 30.3, 29.49; IR, RD-KBr (cm^−1^): 3466, 3008, 2960, 2918, 2850, 1745, 1541, 1466, 1433, 1387, 1240, 1192, 1109, 1059, 891, 783, 723.

*2-(Hexadecyloxy)-2-oxoethanaminium methanesulfonate* (**3g**). White solid; Mp. 85–86 °C; ^1^H-NMR (δ, CD_3_OD): 8.32 (ws), 4.24 (t, *J* = 6.7 Hz, 2H), 3.81 (s, 2H), 2.74 (s, 3H), 1.69 (qi, *J* = 7.0 Hz, 2H), 1.28 (m, 26H), 0.89 (t, *J* = 7.0 Hz, 3H); ^13^C-NMR: 166.4, 77.4, 76.6, 65.5, 39.0, 38.9, 37.7, 31.6, 31.0, 28.8, 28.7, 28.6, 28.6, 28.5, 28.3, 27.5, 24.92, 24.8, 21.7, 12.7; IR, RD-KBr (cm^−1^): 3390, 3024, 2924, 2914, 2850, 1747, 1514, 1473, 1439, 1387, 1242, 1174, 1051, 918, 864, 715.

*2-(Octadecyloxy)-2-oxoethanaminium methanesulfonate* (**3h**). White solid; Mp. 72 °C (dec.); ^1^H-NMR (δ, CD_3_OD): 8.36 (ws), 4.24 (t, *J* = 6.7 Hz, 2H), 3.81 (s, 1H), 3.75 (s, 1H), 2.74 (s, 3H), 1.70 (qi, *J* = 7.0 Hz, 2H), 1.28 (m, 30H), 0.89 (t, *J* = 6.9 Hz, 3H); ^13^C-NMR: 166.8, 77.4, 76.9, 76.5, 65.4, 61.1, 38.9, 38.8, 37.6, 31.6, 31.0, 28.8, 28.7, 28.6, 28.5, 28.4, 28.3, 27.5, 24.9, 24.8, 21.7, 12.68; IR, RD-KBr (cm^−1^): 3388, 3088, 2918, 2850, 1747, 1514, 1473, 1383, 1240, 1176, 1051, 912, 781, 715.

*(S)-1-(Benzyloxy)-1-oxopropan-2-aminium methanesulfonate* (**3i**). Brown oil; ^1^H-NMR (δ, CD_3_OD): 7.38 (s, 5H), 5.26 (s, 2H), 4.08 (q, *J* = 7.3 Hz, 1H), 2.74 (s, 3H), 1.58 (d, *J* = 7.3 Hz, 3H); ^13^C-NMR: δ 169.2, 134.2, 128.3, 128.2, 127.9, 77.4, 76.9, 76.5, 67.6, 38.3, 15.2, 15.1; IR, RD (cm^−1^): 3431, 3032, 2954, 2843, 22748, 1747, 1525, 1498, 1456, 1329, 1213, 1151, 1113, 1038, 958, 910, 777.

*(S)-1-(Ethyloxy)-1-oxopropan-2-aminium p-toluenesulfonate* (**3j**). White solid; Mp. 218–220 °C; ^1^H-NMR (δ, CD_3_OD): 8.33 (ws), 7.78 (d, *J* = 8.2 Hz, 2H), 7.29 (d, *J* = 8.2 Hz, 2H), 4.25 (q, *J* = 7.0 Hz, 2H), 3.61 (q, *J* = 7.1 Hz, 1H), 2.36 (s, 3H), 1.74 (d, *J* = 7.2 Hz, 3H), 1.18 (t, *J* = 7.0 Hz, 3H); ^13^C-NMR: 170.3, 140.9, 139.8, 128.0, 124.9, 77.4, 76.9, 76.5, 76.5, 19.8, 14.6; IR, RD-KBr (cm^−1^): 3469, 3066, 2951, 2851, 1749, 1518, 1460, 1203, 1126, 1113, 1043, 1014, 814, 688, 567.

*(S)-1-(Octyloxy)-1-oxopropan-2-aminium p-toluenesulfonate* (**3k**). White solid; Mp. 203–205 °C; ^1^H-NMR (δ, CD_3_OD): 8.32 (ws), 7.73 (d, *J* = 8.2 Hz, 2H), 7.22 (d, *J* = 8.1 Hz, 2H), 3.95 (qi, *J* = 7.3 Hz, 1H), 3.33 (m, 2H), 2.37(s, 3H), 1.54 (d, *J* = 7.3 Hz, 3H), 1.46 (m, 2H), 1.31 (m, 10H), 0.89 (t, *J* = 6.7 Hz, 3H); ^13^C-NMR: 170.4, 141.0, 139.8, 130.2, 128.0, 124.9, 77.4, 76.9, 76.6, 48.1, 47.8, 47.5, 47.2, 46.9, 19.9, 19.96, 14.6; IR, RD-KBr (cm^−1^): 3477, 3066, 2900, 2825, 1749, 1601, 1520, 1462, 1244, 1203, 1163, 1126, 1043, 1012, 860, 814, 688, 656.

*(S)-1-(Decyloxy)-1-oxopropan-2-aminium methanesulfonate* (**3l**). White solid; Mp. 94–95 °C; ^1^H-NMR (δ, CD_3_OD): 8.43 (ws), 4.24 (qd, *J* = 4.5, 2.2 Hz, 2H), 4.05 (q, *J* = 7.2 Hz, 1H), 2.74 (s, 3H), 1.69 (qi, *J* = 7.1 Hz, 2H), 1.57 (d, *J* = 7.3 Hz, 3H), 1.29 (m, 14H), 0.89 (t, *J* = 6.9 Hz, 3H); ^13^C-NMR: 169.1, 77.4, 76.9, 76.5, 65.7, 37.8, 31.0, 28.7, 28.6, 28.5, 28.4, 27.6, 24.9, 21.8, 14.7, 14.6, 12.8; IR, RD-KBr (cm^−1^): 3491, 3059, 2958, 2918, 2852, 1751, 1523, 1470, 1336, 1244, 1205, 1157, 1119, 1043, 1005, 775, 750, 555.

*(S)-1-(Dodecyloxy)-1-oxopropan-2-aminium p-toluenesulfonate *(**3m**). White solid; Mp. 224–225 °C. ^1^H-NMR (δ, CD_3_OD): 8.31 (ws), 7.73 (d, * J* = 8.2 Hz, 2H) 7.22 (d, *J* = 8.0 Hz, 2H), 3.95 (qi, *J* = 7.3 Hz, 1H), 3.33 (m, 2H), 2.37 (s, 3H), 1.54 (d, *J* = 7.3 Hz, 3H), 1.46 (m, 2H), 1.31 (m, 18H), 0.89 (t, *J* = 6.7 Hz, 3H); ^13^C-NMR: 170.4, 140.9, 139.8, 128.1, 124.9, 77.4, 76.9, 76.5, 20.1, 20.0, 14.7; IR, RD-KBr (cm^−1^): 3471, 3068, 2949, 2906, 2829, 1749, 1520, 1205, 1126, 1113, 1043, 1014, 816, 688, 567.

*(S)-1-(Tetradecyloxy)-1-oxopropan-2-aminium methanesulfonate* (**3n**). White solid; Mp. 86–87 °C; ^1^H-NMR (δ, CD_3_OD): 4.24 (qd, *J* = 4.0, 2.2 Hz, 2H), 4.04 (q, *J* = 7.3 Hz, 1H), 2.75 (s, 3H), 1.70 (qi, *J* = 7.0 Hz, 2H), 1.57 (d, *J* = 7.3 Hz, 3H), 1.27 (m, 22H), 0.89 (t, *J* = 7.0 Hz, 3H); ^13^C-NMR: 169.2, 77.4, 76.9, 76.6, 65.9, 38.0, 31.8, 31.2, 28.9, 28.95, 28.9, 28.8, 28.8, 28.6, 28.5, 27.7, 25.0, 21.9, 14.9, 14.8, 13.0; IR, RD-KBr (cm^−1^): 3491, 3145, 2958, 2918, 2850, 1751, 1531, 1470, 1207, 1157, 1120, 1043, 775, 721, 555.

*3-(1H-Indol-3-yl)-1-(octyloxy)-1-oxopropan-2-aminium methanesulfonate* (**3o**). White solid; Mp. 119–121 °C; ^1^H-NMR (δ, DMSO-*d*_6_): 10.99 (s, NH), 8.36 (ws, 3H), 7.46 (d, *J* = 7.8 Hz, 1H), 7.36 (d, *J* = 7.9 Hz, 1H), 7.21 (dd, *J* = 6.8 Hz, *J* = 2.1 Hz, 1H), 7.05 (td, *J* = 6.8, 2.1 Hz, 1H), 6.97 (td, *J* = 6.8, 1.0 Hz, 1H), 4.17 (s, 1H), 4.01 (m, 2H), 3.27 (m,2H), 2.46 (s, 3H), 1.45 (qi, *J* = 6.7 Hz, 2H), 1.20 (m, 10H), 0.85 (t, *J* = 7.1 Hz, 3H); ^13^C-NMR: 167.5, 135.4, 134.4 124.9, 122.9, 119.2, 116.7, 115.9, 109.7, 104.4, 77.4, 76.9, 76.5, 63.8, 50.9, 29.42, 26.8, 26.7 25.9, 24.4, 23.3, 20.3, 12.0; IR, RD-KBr (cm^−1^): 3331, 3062, 2953, 2870, 2854, 1998, 1747, 1522, 1458, 1437, 1362, 1288, 1209, 1039, 947, 781, 737, 557.

*3-(1H-Indol-3-yl)-1-(decyloxy)-1-oxopropan-2-aminium methanesulfonate* (**3p**). White solid; Mp. 124–125 °C; ^1^H-NMR (δ, DMSO-*d*_6_): 11.04 (ws), 8.40 (ws), 7.49 (d, *J* = 7.8 Hz, 1H), 7.39 (d, *J* = 8.0 Hz, 1H), 7.23 (dd, *J* = 6.5,2.4 Hz, 1H), 7.09 (td, *J* = 7.0, 1.0 Hz, 1H), 7.00 (td, *J* = 7.0, 1.0 Hz, 1H), 4.21 (s, 1H), 4.03 (m, *J* = 4.7 Hz, 2H), 3.30 (dd, *J* = 6.4, 4.0 Hz, 2H), 2.47 (s, 3H), 1.46 (qi, *J* = 6.8 Hz, 2H), 1.24 (m, 14H), 0.87 (t, *J* = 6.9 Hz, 3H); ^13^C-NMR: 172.2, 139.1, 129.6, 127.6, 123.9, 121.3, 120.6, 114.4, 109.0, 82.1, 81.7, 81.3, 68.4, 55.6, 34.1, 31.9, 31.8, 31.6, 31.5, 30.6, 29.1, 27.9, 24.9, 16.8; IR, RD-KBr (cm^−1^): 3331, 3068, 2953, 2920, 2848, 2000, 1749, 1524, 1458, 1437, 1362, 1290, 1211, 1173, 1120, 1078, 1038, 947, 837, 779, 746, 557.

*3-(1H-Indol-3-yl)-1-(dodecyloxy)-1-oxopropan-2-aminium methanesulfonate* (**3q**). White solid; Mp. 122–123 °C; ^1^H-NMR (δ, DMSO-*d*_6_): 11.02 (ws), 8.38 (ws), 7.48 (d, *J* = 7.8 Hz, 1H), 7.38 (d, *J* = 8.0 Hz, 1H), 7.23 (dd, *J* = 6.6, 2.3 Hz, 1H), 7.08 (td, *J* = 6.9, 1.0 Hz, 1H), 7.00 (td, *J* = 7.0, 1.0 Hz, 1H), 4.21 (s, 1H), 4.04 (m, *J* = 4.1 Hz, 2H), 3.30 (dd, *J* = 5.8, 1.8 Hz, 2H), 2.47 (s, 3H), 1.48 (qi, *J* = 6.8 Hz, 2H), 1.25 (m, 18H), 0.87 (t, *J* = 7.0 Hz, 3H); ^13^C-NMR: 167.5, 134.4, 124.9, 122.9, 119.2, 116.7, 115.9, 109.7, 104.3, 77.4, 76.9, 76.5, 63.8, 50.9, 29.5, 27.3, 27.2, 27.2, 27.1, 26.9, 26.9, 25.9, 24.4, 23.3, 20.3, 12.1; IR, RD-KBr (cm^−1^): 3331 (ν_NH_), 3068, 3028, 2953, 2920, 2848 (ν_CH_), 2000, 1749 (ν_C=O_), 1595, 1524, 1458, 1437, 1362, 1290, 1213 (ν_SO3_), 1173, 1120, 1078, 1038, 947, 837, 779, 746, 557.

*3-(1H-Indol-3-yl)-1-(tetradecyloxy)-1-oxopropan-2-aminium methanesulfonate* (**3r**). White solid; Mp. 118–119 °C; ^1^H-NMR (δ, DMSO-*d*_6_): 11.05 (ws), 8.38 (ws), 7.48 (d, *J* = 7.7 Hz, 1H), 7.38 (d, *J* = 8.0 Hz, 1H), 7.23 (dd, *J* = 5.9, 2.3 Hz, 1H), 7.09 (td, *J* = 7.0, 1.0 Hz, 1H), 6.99 (td, *J* = 7.0, 2.1 Hz, 1H), 4.22 (s, 1H), 4.02 (m, *J* = 4.4 Hz, 2H), 3.30 (d, *J* = 5.5 Hz, 2H), 2.45 (s, 3H), 1.45(q, *J* = 6.4 Hz, 2H), 1.24 (m, 18H), 0.86 (t, *J* = 6.4 Hz, 3H); ^13^C-NMR: 167.5, 134.3, 124.9, 122.9, 119.2, 116.6, 115.9, 109.6, 104.3, 77.4, 76.9, 76.5, 63.7, 50.8, 29.4, 27.2, 27.1, 27.1, 27.0, 26.7, 26.6, 25.8, 24.3, 23.2, 20.2, 12.0; IR, RD-KBr (cm^−1^): 3331, 3070, 3028, 2953, 2920, 2848, 1998, 1751, 1593, 1524, 1458, 1437, 1362, 1290, 1213, 1173, 1122, 1078, 1038, 947, 837, 779, 744, 555.

*3-(1H-Indol-3-yl)-1-(hexadecyloxy)-1-oxopropan-2-aminium methanesulfonate* (**3s**). White solid; Mp. 120–121 °C; ^1^H-NMR (δ, DMSO-*d*_6_): 11.05(ws), 8.38 (ws), 7.48 (d, *J* = 7.8 Hz, 1H), 7.38 (d, *J* = 8.0 Hz, 1H), 7.23 (dd, *J* = 5.7, 2.3 Hz, 1H), 7.08 (td, *J* = 7.0, 1.0 Hz, 1H), 7.00 (td, *J* = 7.0, 0.9 Hz, 1H), 4.22 (s, 1H), 4.02 (m, *J* = 6.5 Hz, 2H), 3.30 (dd, *J* = 3.3 Hz, *J* = 5.8 Hz, 2H), 2.44 (s, 3H), 1.46 (qi, *J* = 6.5 Hz, 2H), 1.24 (m, 26H), 0.86 (t, *J* = 6.5 Hz, 3H); ^13^C-NMR: 167.5, 134.3, 124.9, 122.9, 119.2, 116.6, 115.9, 109.6, 104.3, 77.4, 76.9, 76.5, 63.7, 50.8, 38.4, 38.1, 37.8, 37.6, 37.5, 37.3, 36.9, 29.4, 28.7, 27.2, 27.1, 27.0, 26.9, 26.8, 25.9, 24.4, 23.2, 20.2, 12.0; IR, RD-KBr (cm^−1^): 3331, 3068, 3028, 2953, 2920, 2848, 1751, 1595, 1524, 1464, 1437, 1362, 1290, 1213, 1173, 1120, 1038, 949, 837, 779, 744, 555.

*3-(1H-Indol-3-yl)-1-(octadecyloxy)-1-oxopropan-2-aminium methanesulfonate *(**3t**). White solid; Mp. 114–116 °C (dec); ^1^H-NMR (δ, DMSO-*d*_6_): 10.99 (ws), 8.36 (ws), 7.46 (d, *J* = 7.8 Hz, 1H), 7.36 (d, *J* = 7.9 Hz, 1H), 7.21 (dd, *J* = 6.8 Hz, *J* = 2.1 Hz, 1H), 7.05 (td, *J* = 6.8, 2.1 Hz, 1H), 6.97 (td, *J* = 6.8, 1.0 Hz, 1H), 4.17 (s, 1H), 4.01 (m, 2H), 3.27 (m, 2H), 2.46 (s, 3H), 1.45 (qi, *J* = 6.7 Hz, 2H), 1.20 (m, 10H), 0.85 (t, *J* = 7.1 Hz, 3H); ^13^C-NMR: 167.5, 1354.5, 134.4 124.9, 122.9, 119.2, 116.7, 115.9, 109.7, 104.4, 77.4, 76.9, 76.5, 63.8, 50.9, 29.42, 26.8, 26.7 25.9, 24.4, 23.3, 20.3, 12.0; IR, RD-KBr (cm^−1^): 3331, 3068, 2920, 2848, 1751, 1597, 1524, 1464, 1377, 1290, 1213, 1173, 1120, 1038, 947, 837, 779, 744, 555.

*3-(1H-Indol-3-yl)-1-(octadecyloxy)-1-oxopropan-2-aminium p-toluenesulfonate* (**3u**). White solid; 92 °C (dec.); ^1^H-NMR (δ, DMSO-*d*_6_): 10.91 (ws), 8.36 (ws), 7.64 (d, *J* = 8.1 Hz, 2H), 7.47 (d, *J* = 7.8 Hz, 1H), 7.38 (d, *J* = 7.9 Hz, 1H), 7.23 (dd, *J* = 9.2, 2.3 Hz, 1H), 7.13 (d, *J* = 7.9 Hz, 2H), 7.08 (t, *J* = 7.2 Hz, 1H), 7.02(t, *J* = 7.2 Hz, 1H), 4.15 (3, 1H), 4.07 (m, 2H), 3.32 (d, *J* = 6.2 Hz, 2H), 2.33 (s, 3H), 1.53 (qi, *J* = 6.4 Hz, 2H), 1.25 (m, 30H), 0.87 (t, *J* = 6.4 Hz, 3H); ^13^C-NMR: 167.7, 142.7, 136.8, 134.8, 126.6, 125.2, 124.0, 123.3, 119.7, 119.6, 117.1, 116.1, 110.0, 109.9, 104.5, 77.4, 76.9, 76.5, 64.3, 51.3, 51.2, 29.9, 27.7, 27.6, 27.5, 27.4, 27.3, 26.4, 23.8, 20.7, 19.4, 12.4; IR, RD-KBr (cm^−1^): 3431, 3269, 3240 (ν_NH_), 2951, 2916, 2848, 1741, 1578, 1504, 1471, 1437, 1346, 1290, 1165, 1126, 1036, 1012, 814, 731, 575, 561.

## 4. Conclusions

The simultaneous esterification and acid-base reaction of unprotected α-amino acids to obtain ionic amino acid esters was demonstrated to be efficiently promoted in a one-pot, solventless approach using a wide range of alcohols and amino acids using MsOH or *p-*TsOH as acid catalysts under microwave irradiation. The proposed versatile protocol may be in principle easily extended to a range of other compounds, paving the way to the facile preparation of related relevant organic compounds of industrial important as surfactants and emulsifiers. Further investigations of these reactions are currently being ongoing in our laboratories.
